# Pulmonary edema following shoulder arthroscopy under general anesthesia with nerve block: An observational study

**DOI:** 10.1097/MD.0000000000023713

**Published:** 2020-12-18

**Authors:** Gang Zhang, Qihai Wan, Xiaoyan Huang, Yunhua Shui, Chunqiong Luo, Li Su, Xue Jiang, Lan Zhang

**Affiliations:** a Department of Anesthesia, Sichuan Provincial Orthopedic Hospital (Chengdu Sports Hospital and Chengdu Research Institute for Sports Injury); b Chengdu University of Traditional Chinese Medicine; c Department of Operation Room, the Third People's Hospital of Chengdu, Chengdu, China.

**Keywords:** shoulder arthroscopy, pulmonary edema, ultrasound, B lines

## Abstract

Shoulder arthroscopy requires a large of irrigation for a better surgical view, leading circulatory overload. This study was performed to prove whether pulmonary edema will be lead by a large of irrigation.

General anesthesia with interscalene block was induced before operation. The primary outcome was ultrasound evaluation of B lines from the time before nerve block to the time 10 hours after operation. The secondary outcomes included oxygenation index, arterial partial pressure of carbon dioxide, visual analogue scale, muscle strength grade.

A total of 93 patients were evaluated. Before surgery, B lines failed to be detected. While the highest total incidence of B lines was 49.4%, occurred at 4 hours after surgery. The highest incidences of severe and moderate pulmonary edema were 3.2% (*P* = .081) and 9.7% (*P* = .002), respectively. B lines were also found on both the affected and healthy side. During operation, the incidence of type 1 respiratory failure was 5.4% (*P* = .023) and that of both type 1 and 2 respiratory failure were 6.5% (*P* = .013). Pain was relieved in 6 hours after surgery (VAS < 3). At 12 hours after operation, the VAS of resting and motion were 4.68 ± 2.27, 6.90 ± 2.43, respectively. While the grade of muscle strength was 4.48 ± 0.51 at 12 hours after operation.

There is a high incidence of pulmonary edema in shoulder arthroscopy, and ultrasound is a convenient tool to evaluate this complication. Pain is relieved in 6 hours after surgery by nerve block. While muscle strength can also recover at 12 hours after surgery.

## 1 Introduction

Shoulder arthroscopy, a minimally invasive surgery procedure, has become a routine way to diagnose and treat a variety of shoulder joint diseases. Compared with traditional surgery, it has obvious advantages such as a faster postoperative recovery and shorter hospital stay.^[1]^ Despite these benefits it still has some shortcomings. For a better surgical view, it requires a large of irrigation fluid to distend the joint cavity, which may lead a serious extravasation to the adjacent soft tissues. It may damage shoulder capsule, leading the irrigation to permeate into the outer space. As a result, it may lead the occurrence of significant edema of face, neck and chest tissues, tracheal compression, upper respiratory tract obstruction, and pulmonary edema. For swelling of adjacent neck, chest, and facial tissues, most patients will be lack of obvious syndrome within 2 days after surgery.^[2]^ However, in severe cases, excessive irrigation will flow into the pharyngolaryngeal and paratracheal space, which can lead upper respiratory obstruction ^[3,4]^ and even pulmonary edema.^[5]^

**Figure 1:**
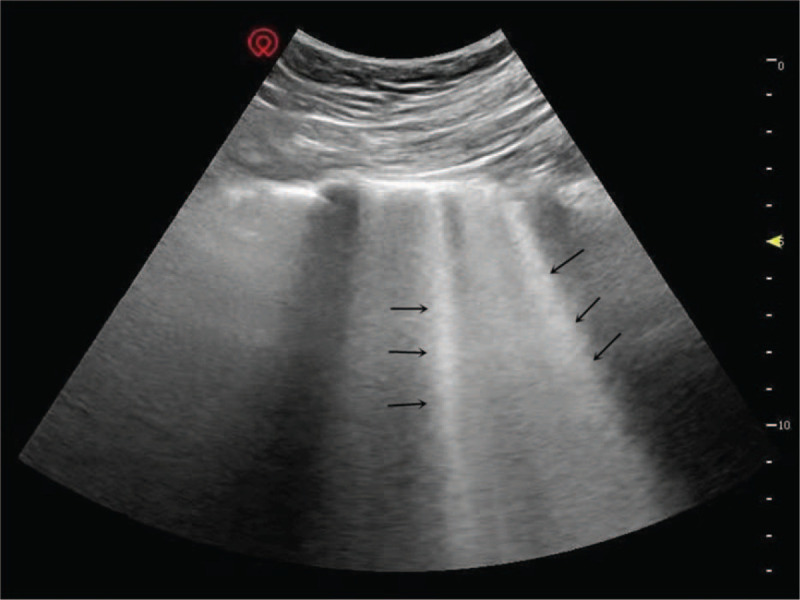
B lines were detected by ultrasound (black arrows).

Complications caused by irrigation have been paid more and more attention.^[6]^ Borgeat reported a case of life-threatening respiratory distress by the large extravasation.^[7]^ Gogia reported a case of airway obstruction for severe neck edema after extubation, and then negative pressure pulmonary edema occurred.^[5]^ In this case, negative-pressure pulmonary edema is directly caused by airway obstruction. However, there is not any report of pulmonary edema caused by overload of circulation in shoulder arthroscopy. This complication caused by excessive flushing fluid has been reported in transurethral prostatectomy ^[8]^ and hysteroscopic surgery.^[9]^ To ensure the surgical vision, shoulder arthroscopy also requires a large amount of irrigation, which may lead an overload of circulation. Structures near the shoulder joint is attached by a large of muscles rich of blood, which is the anatomic basis for absorption into the blood. Before this study, a lot of shoulder arthroscopy had been performed in our hospital. For a better surgical view, shoulder arthroscopy requires a large of irrigation in some patients (more than 30L). After the exclusion of negative pressure pulmonary edema (NPPE), some patients experienced unexplained oxygen desaturation with moist rales in both lungs. B lines could also be detected by ultrasound in different intercostal spaces (Fig. 1). Will the life-threatening pulmonary edema be led by a large of irrigation fluid in shoulder arthroscopy? Ultrasound is a reliable technique for monitoring various pulmonary edema. Compared with X-ray, it has the advantages of no radiation and real-time.^[10]^ The number of B lines (N_B_) from ultrasound also plays important roles in evaluating severity of pulmonary edema.^[11]^ In our hospital, swelling of adjacent tissues and pulmonary edema, have been observed in a number of patients by ultrasound. For ensuring safety, will the ultrasound be a convenient tool to rapidly assess and identify this complication? Consequently, to answer the 2 questions above, we performed this observational study.

## 2 Methods

This study was performed after approval by Research Ethics Committee of Sichuan Provincial Orthopedic Hospital (Chengdu Sports Hospital and Chengdu Research Institute for Sports Injury). Written informed consent was obtained from all the patients before study. It was also registered prior to patient enrollment at Chinese Clinical Trial Registry (http://www.chictr.org.cn; Registration number: ChiCTR1900023793; Principal investigator: Lan Zhang; Date of registration: June 12, 2019). This study adheres to the applicable CONSORT guidelines. Exclusion criteria included pulmonary diseases (chronic pulmonary diseases or pulmonary edema), body mass index ≥35 kg/m^2^, unable to perform brachial plexus nerve block (skin infection, brachial plexus nerve injury), severe liver or kidney disease, abnormal coagulation function. Oxygen saturation, electrocardiogram, noninvasive blood pressure and body temperature were monitored once the patients had been into the operating room. Interscalene brachial plexus block (ISBPB) was performed guided by ultrasound and nerve stimulator before general anesthesia. Local anesthetics (0.33% ropivacaine, 30 ml) were also injected at the back of brachial plexus to provide adequate analgesia and muscle relaxation. Then the radial artery of healthy side was punctured and catheterized to monitor invasive blood pressure. Patients were preoxygenated and anesthesia were induced with sufentanil 0.5μg/kg and propofol 2.5 mg/kg. Neuromuscular blockades were followed by a single infusion of rocuronium 0.6 mg/kg. Intubation was induced and the tidal volume of mechanical ventilation was set to 8 to 10 ml/kg. Patients undergoing shoulder arthroscopy would be evaluated dynamically by pulmonary ultrasouond (Navi U, Wisonic Inc, CN) from the beginning of operation to the time at 10 hours after operation. Monitoring time points included the time before nerve block, the time after surgery (or time in postanesthesia care unit, PACU) and the time at 2, 4, 6, 8, 10 hours after operation (T_be_, T_pa_, T_2_, T_4_, T_6_, T_8_, T_10_). The second, third, fourth and fifth intercostal spaces of the right lung were scanned with convex array probe along the parasternal line, midclavicular line, anterior axillary line and midaxillary line, while the second, third and fourth intercostal spaces of the left lung were scanned along the same anatomic location.

Fluid therapy was performed according to body weight. The normal physiological requirement was 4 ml/kg.h for the first 10 kg, 2 ml/kg.h for the second 10 kg, and 1 ml/kg.h for remaining weight (RW). The formula to calculate the volume of fluid therapy was listed below (T_f+o_: Time for fasting and operation).

$$\text{Volume}\;(\text{ml})=[10\times 4+10\times 2+\text{RW}\;(\text{kg})\times 1]\times {\text{T}}_{\text{f}+\text{o}}\;(\text{h})$$

The primary outcome was continuous number counting of B lines in 10 hours after surgery. B lines from ultrasound were also used as an important diagnostic indicator of pulmonary edema. According to N_B_, patients were classified as 4 groups (non-pulmonary edema: NB ≤ 5; mild pulmonary edema: NB = 6-15; moderate pulmonary edema: NB = 16-29; severe pulmonary edema: NB ≥ 30).^[11]^ While respiratory parameters were also monitored by blood gas analysis (BGA). Oxygenation index (OI) was equal to the ratio of partial arterial O_2_ pressure (PaO_2_) to fraction of inspiration O_2_ (FiO_2_). Other outcomes were muscle strength evaluation and visual analogue score (VAS) during rest and movement in 12 hours after surgery.

### 2.1 Statistical analysis

SPSS 23.0 software (SPSS Inc, Chicago, IL) was used to process and analyze the data. Measurement data in line with normal distribution were expressed as Mean ± SD, and tested by *t* test. Measurement data not coincided with normal distribution were expressed as Median (Q1, Q3), and tested by Wilcoxons Sign Rank Test. Enumeration data were expressed as percentage and tested by Fisher exact test. A value of *P* < .05 was considered statistically significant.

## 3 Results

A total of 93 patients undergoing shoulder arthroscopy were included between June and September 2019 and no patient was excluded. Demographic and surgical data were list in Table 1. The volume of fluid infusion was 831.67 ± 224.96 ml, calculated by weight of the patients.

**Table 1:** Demographic and surgical data.

	Value
Sex (M/F)	45/48
Age (y)	55.73 ± 13.75
BMI (kg/m^2^)	23.33 ± 2.97
Urine (ml)	310.00 ± 167.84
Bleeding (ml)	30.67 ± 15.74
Operation time (min)	118.87 ± 44.16
Anesthesia time (min)	183.16 ± 50.94
Fuid infusion volume (ml)	831.67 ± 224.96
Irrigation (L)	24.55 ± 13.24

**Figure 2:**
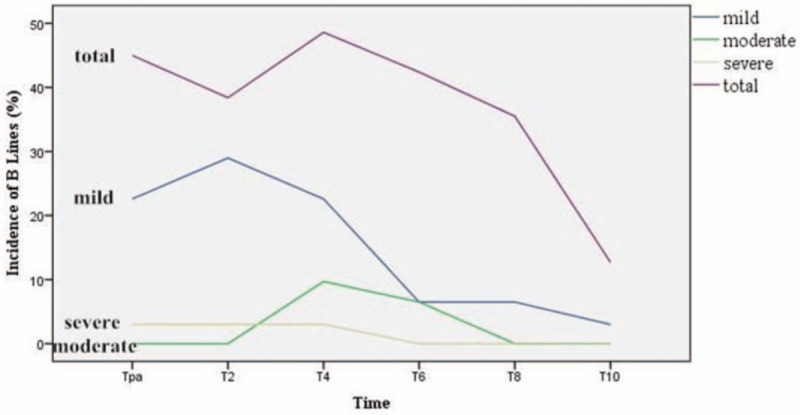
The curves indicated incidence of B lines from the end of surgery to 10 hours after operation.

The highest total incidence of B lines was 49.4%, occurred at 4 hours after surgery. The highest incidence of moderate pulmonary edema was 9.7% (*P* = .002). These incidences above were higher in comparison with that before operation (*P* < .05). The highest incidence of severe pulmonary edema was 3.2% and there was no significant difference when compared with that before operation. However, the value of *P* was 0.081%, which was too close to 0.05%. (Table 2, Fig. 2) A total of 3 patients, unable to deoxygenate, were diagnosed with severe pulmonary edema and should be monitored in PACU. Furosemide was administrated, and all B lines disappeared at the time 4 hours after operation. All patients were sent back to the ward when the OI returned to the preoperative level after deoxygenation.

**Table 2:** Incidence of B lines at different time.

Level/Time	T_pa_ (n = 93)	T_2_ (n = 93)	T_4_ (n = 93)	T_6_ (n = 93)	T_8_ (n = 93)	T_10_ (n = 93)
None	18 (19.7%)	6 (6.4%)	13 (13.9%)	18 (19.7%)	27 (29.0%)	9 (9.7%)
Mild	21 (22.6%)	27 (29.0%)	21 (22.6%)	6 (6.5%)	6 (6.5%)	3 (3.2%)
Moderate	0	0	9 (9.7%)	6 (6.5%)	0	0
Severe	3 (3.2%)	3 (3.2%)	3 (3.2%)	0	0	0
Total	42 (45.5%)	36 (38.6%)	46 (49.4%)	30 (32.7%)	33 (35.5%)	12 (12.9%)

B lines were found on both the affected side and the healthy side. There were no statistical difference in the number of B lines on both sides at T_pa_, T_2_, T_8_, T_10_. The number of B lines on the affected side was larger than that on the healthy side at T_4_, T_6_, and the difference was statistically significant.

Respiratory parameters including OI and arterial partial pressure of carbon dioxide (PaCO_2_) were monitored before and during operation. The patients were divided into type 1 respiratory failure group (T1RF:OI <300 mm Hg and PaCO_2_ <50 mm Hg) and type 2 respiratory failure group (T2RF:OI <300 mm Hg and PaCO2 >50 mm Hg) by OI and PaCO_2_. For saving expenses, we failed to collect the correct breathing data after surgery, such as BGA. All the patients had normal respiratory parameters (OI>300 mm Hg, PaCO2 < 45 mm Hg) before operation. However, during operation the incidence of T1RF was 5.4% (*P* = .023) and that of T1RF + T2RF were 6.5% (*P* = .013). Both the difference above were significant compared with that before surgery (*P* < .05) (Table 3).

**Table 3:** Incidence of respiratory failure before and during operation.

	Before operation (n = 93)	On operation (n = 93)	*P*
T1RF	0	5 (5.4%)	.023
T2RF	0	1 (1.1%)	.316
T1RF + T2RF	0	6 (6.5%)	.013

VAS of resting and motion were 2.94 ± 1.18 and 5.26 ± 1.81 before nerve block. For the benefits from nerve block, pain was relieved in 6 hours after surgery (VAS < 3). At 12 hours after operation, the VAS of resting and motion were 4.68 ± 2.27, 6.90 ± 2.43, respectively. While the grade of muscle strength was 4.48 ± 0.51 at 12 hours after operation.

## 4 Discussion

The primary outcome was the number of B lines in this study. The patients with pulmonary diseases (chronic pulmonary diseases or pulmonary edema) were not involved and B lines could be detected in none of the subjects before surgery. Although numerous minor and major atelectasis, which can also lead B lines, are usual after ISBPB due to some phrenic nerve dysfunction,^[12]^ this probability could be excluded in this study for the B lines were also located in health side rather than in the operation side only where atelectases were led by nerve block.

Early evaluation is the key to the management and treatment of this complication in shoulder arthroscopy. Swan-Ganz catheter, PiCCO or Flowtrac/Vigileo system, as the direct and real-time tools for monitoring circulation overload, were not available to monitor patients for the predictable trauma and high expense. Multiple studies have shown N_B_ could be an indirect index to reflect the circulatory overload.^[12,13]^ The sensitivity and specificity of B lines for the diagnosis of extracascular water (EVLW > 500 ml) were about 90% and 86%, respectively. While the sensitivity and specificity are also high to detect EVLW below 500 ml, indicating ultrasound can be beneficial for early detection of circulation overload.^[14]^ N_B_ in this study to evaluate the severity of pulmonary edema was based on the international experts consensus of pulmonary ultrasound in 2012.^[11]^ Studies have shown that N_B_ changed with the volume of EVLW, which also indicated N_B_ could reflect the severity of pulmonary edema.^[15]^ The advantages to detect B lines by ultrasound are very prominent. First of all, the most obvious feature is convenient and easy. In terms of counting N_B_, with a portable ultrasonic instrument, the beginner can achieve the same effect after just 1 hour of training as the top ultrasonic instrument used by experienced experts.^[16]^ Second, ultrasound also has great advantages of safety, which is radiation-free.^[17]^

Despite the high incidence of this complication, the methods below can help improve patients safety in shoulder arthroscopy. In this study, all of the patients were under operation in the lateral decubitus position, which could increase the risk of extravasation and lead the airway compromise for the gravity.^[18]^ Borgeat and Hynson reported 2 cases of airway compromise in the lateral decubitus position.^[7]^ In this position, the brachial plexus will also be more likely to be injured for continuous tractive effort.^[19]^ The beach-chair position, requiring less irrigation, has many advantages recommended by large number of studies.

1. More visual field: the surgeon will be allowed to easily judge the anatomic structures^[20]^;
2. A wider range of intraoperative shoulder activity: the shoulder joint will be rotated easily for the lack of traction^[21]^;
3. Less bleeding: it provides a lower blood pressure and a better exposure;
4. More convenient for switching to open surgery: when shoulder arthroscopy is not successful, it can be converted to open surgery directly under this position.^[22]^

The irrigation under this surgery is mainly perfused by gravity or automatic pressure device.^[23]^ However, in order to limit the high pressure by the device, irrigation pump powered by gravity is recommended.^[24]^ All the irrigation water were placed on the same level and pumped by gravity in this study.

Isosmotic saline is commonly used in shoulder arthroscopy. However, it may lead a large amount of extravasation into the periarticular tissue space during long time surgery.^[6,25]^ Lo reported the weight gain of patients would increase to 3.95 ± 1.77 kg after surgery.^[26]^ Therefore, the isosmotic saline in this study might increase the risk of fluid extravasation. Hypertonic solution may be a better choice for this surgery. A study reported the weight gain of patients with hypertonic irrigation was 2.25 ± 0.77 kg and the use of hypertonic lavage did not increase operating time.^[27]^ Studies also have shown isotonic or hypotonic extracellular fluid might aggravate the death of chondrocytes in articular cartilage.^[28]^ In contrast, hypertonic lavage fluid had a protective effect on articular cartilage.^[29]^ Animal experiments showed hypertonic fluid could reduce chondrocyte damage and enhance cartilage repairment.^[30]^ Hypertonic solution could not only reduce fluid exudation effectively, but was also beneficial to the recovery of cartilage.

Currently, studies had proved that patients with amount of irrigation less than 20 L were safe. While in patients with symptoms, the total volume of fluid usually ranged from 20 to 36 L.^[31]^ That was about 24.55 ± 13.24 L in this study, which could be a risk leading pulmonary edema. We have also observed the use of furosemide had decreased the incidence of B lines in some patients. Diuretics may reduce the incidence of pulmonary edema by improving blood circulation. However, there is a lack of randomized controlled studies to support it. As a result, it is an important way to decrease the total amount of lavage fluid. In order to limit the volume and improve surgical view, surgeons may consider using electrocautery devices, epinephrine-infused fluids and proper controlled hypotension.^[32]^ However, prolonged surgery may also increase total volume of perfusion. Studies showed the duration of this surgery should be less than 120 minutes.^[24]^ The critical value of operative time to lead airway compromise is about 150 minutes.^[33]^ As a result, risk can be reduced by decreasing the operation time.

Anesthesiologists can also reduce the incidence of complications. At present, general anesthesia combined with ISBPB is recommended for shoulder arthroscopy.

1. Better control of respiration and circulation: endotracheal intubation can provides a secure airway; controlled hypotension can effectively reduce bleeding.^[34]^
2. Lower incidence of complications with general anesthesia: general anesthesia is a safe technology, and the mortality related with anesthesia is only 1 in 300,000.
3. Better management of patients: since the shoulder joint is close to the head, local anesthesia alone may make patients nervous, and the surgical drapes can also cause claustrophobia or other discomfort.^[35]^

General anesthesia can eliminate psychological factors from the patients. The benefits of brachial plexus block include not only excellent muscle relaxation, reduction of opioids but the adequate postoperative analgesia.^[36]^ Therefore, single general anesthesia is not recommended in shoulder arthroscopy.^[37]^

It is also important for timely recognize the change of airway pressure and lung compliance.^[38]^ The tracheal tube cuff deflating test is very helpful to identify edema around the airway. The cuff is deflated in the machine-control breathing mode, and the tidal volume (TV) is recorded. There is leakage around the tracheal tube when TV decreases by more than 100 ml (positive deflating test). If the test is positive, muscle relaxant antagonist is administrated after spontaneous breathing and the tracheal tube can be removed. If there is no leakage around the endotracheal tube (negative deflating test), indicating edema around the airway and extubation is not recommended.

The limitation is that this is an observational study without any intervention, such as the use of fursemi, hormones, or positive end-expiratory pressure ventilation (PEEP). Central venous catheters were not performed, which could be used for the evaluation of blood volume. Future randomized controlled trials also need to be conducted to study interventions to reduce pulmonary edema.

In conclusion, pulmonary edema should not be neglected, for there is a high incidence of this complication in shoulder arthroscopy, which may affect air exchange to decrease oxygenation capacity. It may be effective to reduce the incidence of pulmonary edema by the administration of diuretics, which needs more randomized controlled studies to support. The ultrasound is a convenient tool to rapidly assess and identify this complication. Pain can be relieved in 6 hours after surgery by ISBPB with ropivacaine and muscle strength can also recover at 12 hours after surgery.

## Acknowledgments

We would like to thank Dr. Si Zeng and Dr. Su Li for the statistical methods to the study.

## Author contributions

**Conceptualization:** Gang Zhang.

**Data curation:** Gang Zhang, Qihai Wan, Xiaoyan Huang, Xue Jiang.

**Formal analysis:** Yunhua Shui.

**Funding acquisition:** Li Su, Lan Zhang.

**Investigation:** Gang Zhang, Qihai Wan, Xiaoyan Huang.

**Methodology:** Gang Zhang, Xiaoyan Huang.

**Project administration:** Chunqiong Luo, Lan Zhang.

**Resources:** Chunqiong Luo, Lan Zhang.

**Software:** Lan Zhang.

**Supervision:** Xiaoyan Huang, Yunhua Shui, Li Su.

**Writing – original draft:** Gang Zhang, Qihai Wan, Xiaoyan Huang, Lan Zhang.

**Writing – review & editing:** Gang Zhang, Xiaoyan Huang, Lan Zhang.
